# In the Mouth or in the Gut? Innovation Through Implementing Oral and Gastrointestinal Health Science in Chronic Pain Management

**DOI:** 10.3390/jcm14248812

**Published:** 2025-12-12

**Authors:** Jo Nijs, Ishtiaq Ahmed, Doris Vandeputte, Burel R. Goodin, Tolulope Adetayo, Sébastien Kindt, Matteo Vanroose, Ömer Elma, Elin Johansson, Tine Logghe, Jens Van Akeleyen, Zosia Goossens, Céline Labie, Fabiana Silva, Astrid Lahousse, Eva Huysmans, Rodrigo Núñez-Cortés

**Affiliations:** 1 Pain in Motion Research Group, Department of Physical Therapy, Human Physiology and Anatomy, Faculty of Physical Education & Physical Therapy, Vrije Universiteit Brussel, Jette, 1090 Brussels, Belgium; 2 Brussels Health Campus Jette, PAIN-KIMA, Vrije Universiteit Brussels, Jette, 1090 Brussels, Belgium; 3 PijnPraxis.be Practice for Pain Management, 3970 Leopoldsburg, Belgium; 4 Department of Health and Rehabilitation, Unit of Physiotherapy, Institute of Neuroscience and Physiology, Sahlgrenska Academy, University of Gothenburg, SE-405 30 Gothenburg, Sweden; 5 Department of Clinical Sciences, Faculty of Medicine and Pharmacology, Vrije Universiteit Brussel, 1050 Ixelles, Belgium; 6 Translational Research to Advance Pain Science (TRAPS) Lab, Washington University Pain Center, St. Louis, MO 63110, USA; 7 Department of Psychology, University of Alabama at Birmingham, Campbell Hall, Rm. 415, 1300 University Blvd., Birmingham, AL 35233, USA; 8 Department of Gastroenterology and Hepatology, Universitair Ziekenhuis Brussel, Vrije Universiteit Brussel, Jette, 1090 Brussels, Belgium; 9 School of Allied Health and Exercise Sciences, Faculty of Health, Environment, and Medical Sciences, Bournemouth University, Poole BH12 5BB, UK; 10 Department of Physical Medicine and Rehabilitation, Ziekenhuis Geel, 2440 Geel, Belgium; 11 Brain, Body and Cognition (BBCO), Faculty of Psychology and Educational Sciences, Vrije Universiteit Brussel, 1050 Ixelles, Belgium; 12 Musculoskeletal Rehabilitation Research Group, Department of Rehabilitation Sciences, Faculty of Movement and Rehabilitation Sciences, KU Leuven, 3001 Leuven, Belgium; 13 Rehabilitation Sciences Post Graduation Program, Central Campus, Universidade Federal de Ciências da Saúde de Porto Alegre, Porto Alegre 90050-170, RS, Brazil; 14 Research Foundation—Flanders (FWO), 1000 Brussels, Belgium; 15 Department of Physical Medicine and Physiotherapy, University Hospital Brussels, Jette, 1090 Brussels, Belgium; 16 Department of Physical Therapy, Faculty of Medicine, University of Chile, Santiago 8380286, Chile

**Keywords:** chronic pain, oral dysbiosis, gut dysbiosis, pharmacomicrobiomics, nutrition, sleep management, stress management

## Abstract

Recent scientific advances point towards an important role for oral and gastrointestinal health in people with chronic pain. Poor oral health (e.g., periodontitis, tooth loss) is observed in subgroups of the chronic pain population, including abdominal pain, low back pain, fibromyalgia, and rheumatoid arthritis. In addition to poor oral health, studies have also revealed altered intestinal microbiota compositions in various types of chronic pain, including people with chronic low back pain, knee osteoarthritis, visceral pain, fibromyalgia, tinnitus, and migraine. While overweight/obesity contributes to the likelihood of gut dysbiosis, normal-weight individuals with chronic pain also more often present with poor gut health. Both gastrointestinal and oral health problems (e.g., periodontitis, tooth loss) are increasingly recognized across multiple chronic pain conditions, including abdominal pain, low back pain, fibromyalgia, and rheumatoid arthritis. This perspective paper provides an overview of the requirements for integrating oral and gastrointestinal health in chronic pain management. First and foremost, oral and gastrointestinal health issues need to be recognized as common chronic pain comorbidities that require tailored treatment. Next to recognition of the issue, individuals seeking care for chronic pain need to be screened routinely for these oral and gastrointestinal comorbidities. In terms of management, the following options are suggested: (1) providing oral and gastrointestinal health science education; (2) considering the possible interplay between the gut microbiome and drug treatment (including polypharmacy); (3) expanding the importance of dietary interventions; and (4) considering the potential interplay with other lifestyle factors (e.g., chronic insomnia, overweight/obesity, depression and anxiety). To inform the implementation of these suggestions, longitudinal cohort studies investigating the role of oral and gastrointestinal health in people with chronic pain, as well as studies exploring possible (modifiable) factors that affect the oral and/or gut microbiome, are needed. This includes the bidirectional interplay between the gut microbiome and drugs commonly prescribed to patients with chronic pain. Likewise, adequately powered and controlled clinical trials evaluating the effectiveness of possible treatments for oral and/or gastrointestinal comorbidities in people with chronic pain represent another research priority. Such randomized clinical trials can not only examine the possible causal link between poor oral/gut health and treatment outcomes, but also inform the development of new, innovative ways to improve care for people with chronic pain.

## 1. Introduction

With a global prevalence of 20–30% [1], chronic pain is the most prevalent disease worldwide, leading to substantial disability and enormous societal costs [2]. The pathophysiology of chronic pain entails low-grade systemic inflammation [3,4] and central (nervous system) sensitization [5]. Although chronic pain is recognized as a disease by the World Health Organization (i.e., code MG30 in ICD11 [6,7]), its treatment remains challenging. In any chronic condition, identifying perpetuating factors is crucial for guiding treatment strategies and developing targeted interventions. A growing body of evidence indicates that lifestyle factors such as physical (in)activity, stress, poor sleep, unhealthy diet, and smoking are associated with chronic pain across all age categories [8,9,10,11]. Chronic pain is also associated with decreased life expectancy [12,13], partly due to excess mortality from cancer and cardiovascular disease, and/or misuse of opioid analgesics [13,14,15]. The common ground between adverse lifestyle factors, decreased life expectancy, and chronic pain may be low-grade, systemic inflammation, which is evidently linked to chronic pain and predisposes individuals to the development of life-threatening diseases such as cancer and cardiovascular illness [16]. Hence, behavioral lifestyle factors are increasingly recognized as perpetuating factors for chronic pain, fueling the evidence for a patient-centered multimodal lifestyle approach as the best evidence-based treatment for chronic pain [17,18].

Recent scientific advances point towards an important role of gastrointestinal and oral health in the low-grade, systemic inflammation observed in people with chronic pain. Regarding gastrointestinal health, studies revealed altered intestinal microbiota compositions (dysbiosis) in various types of chronic pain, including chronic low back pain [19], knee osteoarthritis [20], visceral pain [21], fibromyalgia [22], and migraine [23]. In addition to poor gastrointestinal health, poor oral health is also found in different subgroups of the chronic pain population [24], including those with abdominal pain [24], low back pain [25], fibromyalgia [26], and rheumatoid arthritis [27]. Despite growing evidence, gastrointestinal and oral health are often overlooked, and translation into routine clinical practice remains limited, potentially due to the absence of practical frameworks for clinical application in prior reviews. In view of current evidence-based treatment guidelines advocating a multimodal lifestyle approach as first-line treatment for patients with chronic pain [18], this narrative perspective synthesizes current evidence on oral and gastrointestinal health in chronic pain and discusses how common comorbidities and medication exposure (including polypharmacy) may shape microbiome profiles and related inflammatory pathways. To facilitate clinical translation, this perspective presents a practical framework for systematically assessing and integrating oral and gastrointestinal health, alongside comorbidities and medication exposure, into evidence-based multimodal treatment for people with chronic pain, thereby broadening potential therapeutic targets to reduce systemic inflammation and improve patient outcomes.

## 2. Search Strategy and Selection Criteria

Articles for this narrative perspective review were identified by searching PubMed using the terms “gut microbiome”, “oral microbiome”, “dysbiosis”, “chronic pain”, and “systemic health” up to August 2025. Articles were selected based on their relevance to the key arguments of this perspective. Additional papers were identified from the reference lists of retrieved articles and through forward citation tracking of key publications. Only papers published in English were reviewed. Given that this work was conceived as a narrative rather than a systematic review, we did not include a quantitative accounting of records retrieved, a detailed screening protocol, or a formal risk-of-bias assessment.

## 3. The Altered Gut Microbiome in People with Chronic Pain

Arguably, one of the most significant scientific medical/physiological advancements of the past decade represents the establishment of the importance of the gut microbiome in maintaining (or disturbing) human health. Therefore, the gut has often been described as the “second brain,” owing to its extensive enteric nervous system and bidirectional communication with the central nervous system [28], and the gut microbiome has been referred to as the “second genome” of the human body [29,30]. A cross-sectional metagenomic profiling study of individuals from four countries identified three gut enterotypes: a *Bacteroides*-dominated type linked to Western, protein- and fat-rich diets; a *Prevotella*-dominated type associated with fiber-rich diets; and a *Ruminococcaceae*-dominated type with intermediate features [31]. Although not discrete, these enterotypes represent “densely populated areas in a multidimensional space of community composition”, and were further supported by the Flemish Gut Flora Project, which showed that they are shaped by age, diet, medication, and stool consistency [32]. These compositional differences may, in turn, influence gut-immune interactions and inflammatory tone. Components of the microbiome affect the immune composition of the gut through the maturation and differentiation of immune cells and the activation or dampening of inflammation [33]. Indeed, the microbiome produces microbial components and metabolites recognized by the innate immune system (e.g., via toll-like receptors), potentially leading to inflammation [33]. In the case of dysbiosis, which is often present in people with overweight/obesity or even in individuals of normal weight with an unhealthy diet/lifestyle, bacterial products can leak across the gut epithelium (“leaky gut”) and reach organs through the systemic circulation, thereby causing inflammation that may contribute to pain sensitization and persistence [34,35].

Such ‘organs’ include the joints, with the term “gut joint axis” coined to explain the interlinked pathway that involves the gastrointestinal microbiome, immune responses, and joint pathophysiology (e.g., osteoarthritis) [33]. Lipopolysaccharides are cell wall components produced by the microbiota, more specifically, the outer membrane of Gram-negative bacteria, that activate the innate immune system. Consequently, activated immune cells, such as macrophages and neutrophils, bind to the toll-like receptor 4 complex, eliciting inflammatory pathways in cartilage and synovium [33]. Moreover, such lipopolysaccharides make their way from the “leaky gut” to the systemic circulation and induce systemic inflammation [33]. This is likely to occur in people with knee osteoarthritis, among others: a large population-based cohort of people with knee osteoarthritis found a significant association between *Streptococcus* species abundance in stool samples, knee pain severity, and knee inflammation, and these links were also replicated in an independent cohort [20]. These findings were independent of smoking, alcohol consumption, oral medication use, and BMI [20], suggesting an interplay between gastrointestinal health, knee inflammation, and knee pain in people with knee osteoarthritis, a notion underscored by the observation that *Streptococcus* species abundance was significantly associated with knee joint effusion severity [20].

Beyond those with osteoarthritis, there is growing evidence supporting gut dysbiosis in individuals with chronic pain. Systematic reviews and meta-analyses have reported altered gut microbiota composition across different types of chronic pain conditions [36,37], including visceral pain [21,38,39,40], fibromyalgia [22,37], and migraine [23]. Additionally, observational studies and scoping reviews indicate similar patterns in neuropathic pain [41], cancer-related pain [42,43], and chronic tinnitus [44]. Moreover, Mendelian randomization analyses have suggested potential causal associations between gut microbiota and intervertebral disc degeneration, low back pain, and sciatica [45]. A systematic review with meta-analyses revealed decreased gut microbiome diversity (i.e., fewer observed species, lower Shannon index, and phylogenetic diversity) in people with chronic pain compared to pain-free controls [37]. The gut microbiome of people with chronic pain showed lower relative abundance of the *Lachnospiraceae* family, genus *Faecalibacterium* and *Roseburia*, species of *Faecalibacterium prausnitzii* and *Odoribacter splanchnicus*, as well as higher abundance in *Eggerthella* species [37]. Another systematic review found that patients with migraine, compared to controls, have reduced species richness and evenness, and a distinct bacterial composition in the gut microbiome, suggesting an imbalance (dysbiosis) that may lead to reduced gut barrier function, making them more susceptible to increased gut permeability [23]. These observations fit into the evidence linking migraine to gastrointestinal symptoms, such as nausea, irritable bowel syndrome, colitis, and constipation. The specific microbial profile that differentiates people with migraine from healthy individuals is potentially characterized by an overabundance of pro-inflammatory bacteria and/or depletion of protective microbes [23] known for barrier support and anti-inflammatory activity [46]. Similarly, a recent systematic review reported a pro-inflammatory cytokine profile associated with an increased abundance of *Bacteroidetes* and decreased abundance of *Firmicutes* in irritable bowel syndrome [47], supporting the role of gut microbiota composition in visceral pain through the promotion of pro-inflammatory responses. Moreover, systematic reviews highlight the frequent co-occurrence of irritable bowel syndrome and fibromyalgia [48], along with shared microbial features such as reduced abundance of *Bifidobacteriaceae* and *Ruminococcaceae*, further underscoring the overlap between gut dysbiosis, inflammation, and chronic pain [38,49,50].

## 4. The Role of Comorbidities in Gut Dysbiosis in Chronic Pain: Awakening the Gut Feeling

Evidence suggests that obesity is common among individuals with chronic pain, and pain complaints are common in obese individuals [51,52]. Excess adiposity contributes to chronic low-grade systemic inflammation through the release of pro-inflammatory cytokines [53,54]. Obesity is also linked to alterations in gut microbiome composition [55] that may compromise gut barrier integrity, facilitating the translocation of microbiota-derived mediators into systemic circulation. These mediators may contribute to neuroinflammation through the activation of blood–brain barrier cells, microglia, and infiltrating immune cells, thereby triggering and perpetuating central sensitization [56]. While overweight/obesity contributes to the likelihood of gut dysbiosis, people with average weight and chronic pain can also present with poor gut health. For instance, cross-sectional studies found altered gut microbiota composition in people with overweight/obese and low back pain [57], but different intestinal bacterial microbiota composition has also been identified in people with chronic low back pain who are non-elderly, non-obese, and without any other serious chronic diseases [19].

Similar to chronic pain, sleep disorders such as chronic insomnia are also associated with changes in the gut microbiome [58]. Moreover, sleep-related comorbidities are highly prevalent in people with chronic pain, with research findings suggesting a bidirectional relationship [59,60] and poor sleep triggering or perpetuating inflammation [61,62]. Sleep deprivation alters appetite-regulating hormones [58,63,64,65] by reducing leptin, which suppresses appetite, and increasing ghrelin, which stimulates hunger [58,63,64,65]. This hormonal shift leads to increased appetite, snack consumption, and food intake, particularly for high-calorie, fatty foods [63]. At the same time, sleep loss can reduce energy expenditure due to fatigue and lower physical activity. Together, these effects promote weight gain and obesity, especially in environments with easy access to calorie-rich foods, which could lead to gut dysbiosis [34]. In turn, such dysbiosis may further disrupt sleep through the gut–brain axis by altering the production of microbial metabolites [66], suggesting a bidirectional relationship between sleep and gut microbiome function. A systematic review found reduced gut microbiome diversity in people with chronic insomnia, with lower abundance of anti-inflammatory genera such as *Faecalibacterium* and *Lachnospira*, and higher abundance of taxa often associated with pro-inflammatory profiles, including *Actinobacteria*, *Streptococcus*, *Clostridium*, *Blautia*, and *Holdemanella* [67]. To some extent, insomnia severity was also negatively related to the abundance of *Faecalibacterium* and *Lachnospira*, and positively related to *Blautia* [67].

In a cross-sectional study of post-menopausal women with chronic insomnia, the gut microbiome profile differs between objective and paradoxical insomnia (where subjective and objective sleep assessments diverge), with the former showing prominence in the *Coriobacteriaceae* family, including *Collinsella* and *Adlercreutzia,* along with *Erysipelotrichaceae*, *Clostridium*, and *Pediococcus,* and the latter mainly characterized by *Bacteroides*, *Staphylococcus*, *Carnobacterium*, *Pseudomonas*, along with *Odoribacter* [68]. In another cross-sectional study of older adults, gut microbiota richness shows a robust positive association with objective sleep quality, independent of subjective sleep quality and demographics, lifestyle, and health covariates [69]. *Bacteroidetes*, *Ruminococcus*, and *Veillonella*, which are linked to short-chain fatty acid production and anti-inflammatory potential, are associated with better objective sleep quality, whereas *Collinsella* and *Holdemania*, typically linked to pro-inflammatory profiles, are associated with poorer subjective sleep quality in older adults [69]. Importantly, research findings suggest that insomnia-induced inflammatory responses are regulated by the gut microbiota and their metabolites [58].

Psychological distress, including depression, anxiety, and stress, is strongly associated with chronic pain, with meta-analyses indicating that approximately 40% of chronic pain patients experience clinically significant depression and anxiety [70]. Similarly, a systematic review reported that psychological distress disrupts the gut microbiome, increasing pro-inflammatory taxa like *Enterobacteriaceae* and *Desulfovibrio* and reducing short-chain fatty acid-producing bacteria such as *Faecalibacterium*, which heightens inflammation and worsens symptoms via altered tryptophan metabolism and impaired serotonin production [71,72]. Dysregulated hypothalamic–pituitary–adrenal axis signaling, prevalent in chronic pain and psychological distress, elevates cortisol and inflammatory mediators, increasing intestinal permeability (“leaky gut”) and allowing Gram-negative bacteria to translocate, driving chronic neuroinflammation and perpetuating pain sensitivity [37,71]. Conversely, oral and gut dysbiosis exacerbates psychological distress and pain by elevating cytokine production and disrupting vagus nerve signaling, establishing a bidirectional relationship [73,74,75].

## 5. Poor Oral Health in People with Chronic Pain

**Figure 1:**
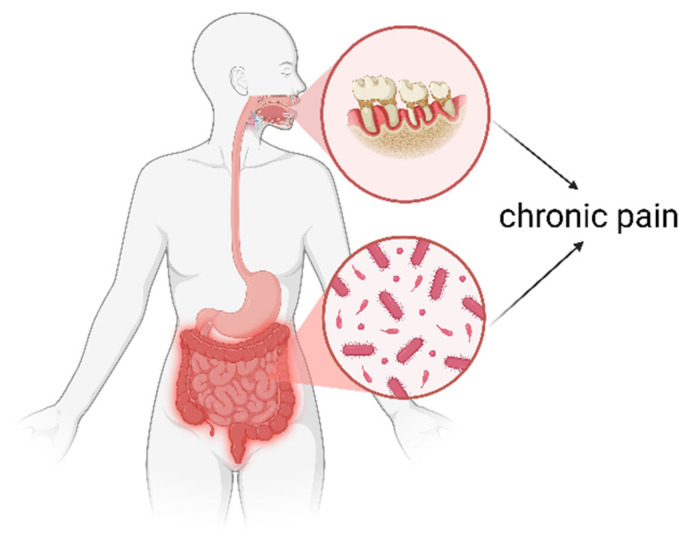
Poor oral and gastrointestinal health are each linked to chronic pain. The figure illustrates periodontitis as an example of poor oral health, and gut dysbiosis as an example of poor gastrointestinal health, both of which are associated with chronic pain.

In addition to poor gastrointestinal health, poor oral health in adults is also associated with chronic pain [25,76] (Figure 1). A population-based study in the United States found a significant link between chronic pain and tooth loss, independent of ethnicity, lifestyle determinants, and immune-mediated inflammatory diseases such as rheumatoid arthritis [76]. In addition, a prospective cohort study in 141 older patients with lumbar degenerative disorders found that the severity of periodontitis is associated with a higher incidence of intervertebral disc degeneration, endplate changes and clinical outcomes, suggesting a mouth–gut–disc axis [25]. Rheumatoid arthritis is also associated with tooth loss and periodontitis [27], and people with periodontitis have higher risks of developing fibromyalgia [26]. Conversely, in patients with fibromyalgia who never had periodontitis before their diagnosis, the risk of developing periodontitis is also larger, suggesting a bidirectional association [26]. Another observational study found low self-reported oral health scores to be correlated with higher pain scores in women with migraine, abdominal, and body pain [24]. Both pain scores and the presence of chronic pain were associated with higher relative abundance of oral pathobionts such as *Gardnerella*, *Lancefieldella*, and *Mycoplasma salivarius* [24]. A recent case–control study found that in individuals with irritable bowel syndrome, obese participants experienced the highest pain responses to a test solution, which were associated with differences in specific bacterial genera and families [77], suggesting that oral microbial composition may also contribute to pain sensitivity. Although preliminary evidence suggests associations between oral microbiome/oral health and pain, the evidence base remains sparse and is predominantly cross-sectional. Moreover, direct evidence linking oral dysbiosis to low-grade systemic inflammation or central sensitization in people with chronic pain is currently unavailable and represents an important research priority.

Theoretically, oral pathobionts can trigger or perpetuate low-grade systemic inflammation in people (transitioning to or) with chronic pain. This idea is supported by observations of close links between poor oral health and circadian desynchronization [78], sleep disorders [79,80], and psychological disorders [81,82], which in turn can trigger or perpetuate inflammation [61,62]. Recent case–control studies revealed that depression and anxiety can alter the oral microbiome, increasing taxa like *Neisseria species* and *Prevotella nigrescens* while decreasing diversity, promoting periodontitis, and contributing to systemic inflammation along the microbiota-gut–brain axis [81,82]. Oral drug use can also contribute to poor oral health in people with chronic pain. For instance, proton pump inhibitors not only alter gut microbiome composition [83]; they can also cause oral microbiota alterations [84,85].

Collectively, the evidence suggests that dysbiosis of the oral and gut microbiota can trigger or perpetuate low-grade systemic inflammation through barrier dysfunction and immune activation, thereby perpetuating central sensitization. Common comorbidities (e.g., chronic insomnia, overweight/obesity, psychological disorder) and medication exposures (e.g., proton pump inhibitors) further destabilize the microbiome–immune axis. This self-perpetuating loop makes oral and gut health legitimate targets within multimodal pain care: addressing sleep, diet, oral hygiene, psychological disorders, and medication burden can help restore microbial and immune homeostasis, thereby reducing inflammatory tone and interrupting the pain–inflammation cycle.

## 6. Lifestyle Medicine 2.0 for Chronic Pain: Implementing Gastrointestinal and Oral Health Science

### 6.1. Evaluation of Oral and Gut Health in All Patients with Chronic Pain

**Figure 2:**
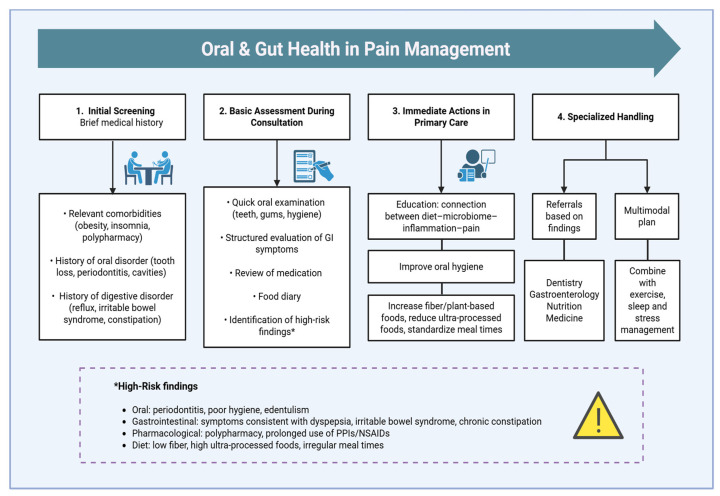
Implementing oral and gastrointestinal health science into chronic pain management: recommendations for assessment of patients with chronic pain. **Abbreviations**: GI: Gastrointestinal; PPI: Proton Pump Inhibitor; NSAID: Nonsteroidal Anti-inflammatory Drug.

In view of the available evidence supporting poor oral and gastrointestinal health in at least a subgroup of the chronic pain population, and in line with the global move towards precision pain medicine, it seems warranted to recognize oral and gastrointestinal health issues as common chronic pain comorbidities that require tailored management/treatment to interrupt or mitigate the dysbiosis–inflammation–pain cycle. This implies that all patients with chronic pain should be screened for possible oral and gastrointestinal health issues. For screening of gastrointestinal health issues in patients with chronic pain, it is proposed to use a structured evaluation of gastrointestinal symptoms (e.g., using instruments such as the *Gastrointestinal Symptom Rating Scale* or the emerging self-reported *Dysbiosis Risk Questionnaire*, which aims to screen for dysbiosis risk based on dietary, antibiotic exposure, and symptom data but remains preliminary in validation), which allows patients to report common symptoms in a standardized manner and assists clinicians in identifying those who may require further assessment [86,87]. The questionnaire data can also assist standard gastrointestinal health history taking (Figure 2). For screening oral health issues in patients with chronic pain, oral inspection together with oral health history taking can be an initial step prior to possible referral to a dentist. Additionally, using food diaries to assess dietary intake can be useful for both oral and gastrointestinal health assessment.

### 6.2. Oral Medication Intake (History), Including Polypharmacy

As part of a comprehensive assessment of oral and gastrointestinal health in patients with chronic pain, it is recommended to question patients with chronic pain regarding oral medication intake, including polypharmacy (Figure 2). For instance, proton pump inhibitors are among the most widely used over-the-counter drugs to treat gastro-esophageal reflux and prevent gastric ulcers, with the gut microbiome composition of proton pump inhibitor users being profoundly different from that of non-users [83,88], mainly due to a strong increase in *Lactobacilli* abundance, driven by *Streptococcus* species. Histamine-2 receptor antagonists also disrupt the gut microbiome but to a lesser degree [88]. Proton pump inhibitors alter the gut microbiota composition by promoting oral-originated *Streptococcus* translocation into the gut [89]. In addition to altering the gut microbiota, proton pump inhibitors can cause hypomagnesemia, particularly with higher doses or prolonged use [90]. Reduced intestinal magnesium absorption, influenced by co-medications and microbiome composition, is particularly concerning in chronic pain patients frequently exposed to polypharmacy affecting electrolyte balance [90]. Another potential over-the-counter medication frequently taken by patients with chronic pain is nonsteroidal anti-inflammatory drugs (NSAIDs), which also affect the gut microbiome composition [91].

Polypharmacy, defined as the concomitant use of ≥5 medications, is the rule rather than the exception among persons living with chronic pain: 71.4% of Canadians and 89.5% of Germans report polypharmacy [92,93], while up to 26% and 49% report at least sometimes excessive polypharmacy (i.e., concurrent use of ≥10 medications [94]). Receiving conservative treatment for chronic pain, such as physical or psychological pain therapy, relates to lower chances of excessive polypharmacy [92], underscoring the need for implementing evidence-based treatments. Polypharmacy, implying multiple drugs active in the central nervous system, is associated with worse pain control, and for patients with daily pain in combination with anxiety, pain was best reduced with one medication or any drug combination without opioids or benzodiazepine (for instance, by using gabapentinoids or serotonin noradrenaline reuptake inhibitors) [95]. Likewise, anxiety in patients with chronic pain was best reduced with combinations other than opioids or benzodiazepines, for instance, through using selective serotonin reuptake inhibitors [95].

Polypharmacy interacts bidirectionally with the human microbiome, as drugs can alter gut microbial diversity and metabolic function, sometimes with effects that persist for years after discontinuation [96]. These alterations accumulate with repeated or combined drug exposures, and polypharmacy is associated with increased abundance of pathobionts, reduced short-chain fatty acid metabolism, and elevated antimicrobial resistance potential [97,98,99]. Conversely, the gut microbiome can enzymatically modify drugs, influencing their absorption, distribution, metabolism, and excretion, thereby affecting drug efficacy and toxicity [100,101,102]. For example, the microbiome can modulate the effectiveness of cancer immunotherapies and the metabolism of psychotropic drugs [100,103], such as duloxetine, which binds to several metabolic enzymes, alters microbial metabolite secretion [104], and is bioaccumulated within bacterial cells without chemical modification. In contrast, melatonin appears beneficial to gut health [105], exerting anti-inflammatory and potentially protective effects [105]. Current large-scale metagenomic and cohort studies confirm that both current and past medication use are major determinants of gut microbiome structure and function, with clinically relevant implications for disease risk, therapeutic efficacy, and adverse events [96,97,99,106,107].

Although this is an exciting new area of science, with the study of the interactions between drugs and bacteria still in its infancy [104], there are currently no large-scale, well-controlled clinical trials directly assessing the impact of medication-induced microbiome changes on chronic pain outcomes. Existing evidence, derived from systematic reviews, Mendelian randomization studies, and small randomized controlled or observational trials, indicates that commonly used analgesics such as NSAIDs and opioids can alter gut microbiota composition, potentially influencing pain persistence and treatment efficacy [108,109,110,111,112]. However, these studies are limited by small sample sizes, short follow-up durations, and heterogeneous outcome measures [109,111], underscoring the need for rigorous, longitudinal research. Nonetheless, co-administration of gut-active supplements (e.g., pro- and prebiotics), combination treatments using dietary interventions to improve gut health as a cornerstone, and strategic polypharmacy may serve as promising and clinically viable approaches for controlling pharmacomicrobiomics interactions [106] in patients with chronic pain.

### 6.3. Oral and Gut Health Science Education

**Figure 3:**
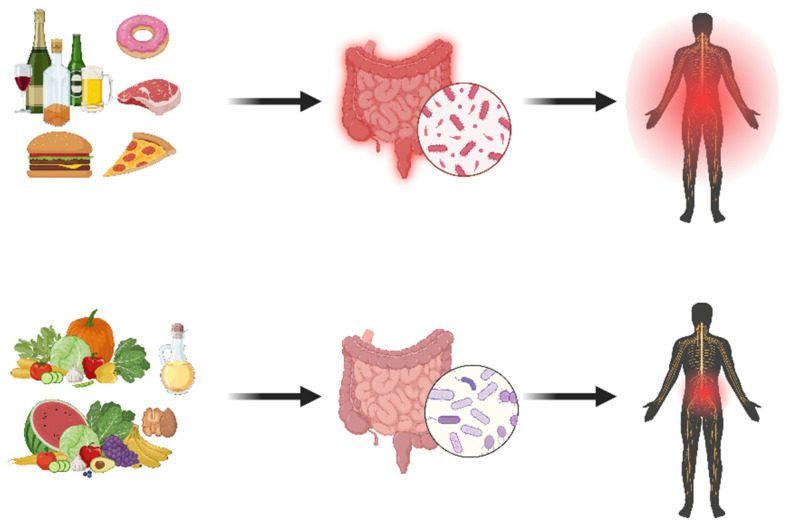
Illustration to explain to patients with chronic pain the connections between an unhealthy diet, gut dysbiosis, chronic low-grade inflammation, and pain severity (**upper part**) versus a healthy diet relating to a healthy gut microbiome and less pain (**lower part**).

Once oral and/or gastrointestinal health issues are identified in a patient with chronic pain, informing the patient about the potential role of improving oral and/or gastrointestinal health can be the first step towards implementing oral and gastrointestinal health science in pain practice. Such education is important for patients to understand the link between disrupted oral and gastrointestinal health and chronic pain, and to become motivated to implement oral and/or gut science (for instance, through improving oral and/or gut health) within an evidence-based multimodal lifestyle approach for managing chronic pain [113]. Educating patients with chronic pain about the role of oral and/or gut health should take clinicians no more than a few minutes and can involve explaining a few general principles about improving their quality and timing of diet to improve their gut’s health (and subsequently potentially improving their pain, as illustrated in Figure 3). The latter is essential not only for general health benefits (e.g., long-term decreased risks of developing cancer, dementia, depression, cardiovascular disease, diabetes), but also for decreasing chronic, low-grade systemic inflammation and subsequent decreased nervous system sensitivity (and hence experiencing less pain), feeling more energetic, less anxious, and less depressed. A similar approach can be advocated in people with chronic pain who have poor oral health (e.g., tooth loss, periodontitis). However, studies examining the added value of such oral health science education and gut health science education to current evidence-based pain science education programs for people with chronic pain are currently unavailable and require empirical testing.

### 6.4. Expanding the Importance of Dietary Interventions

With diet being the first-line treatment for optimizing both oral and gut health, the importance of diet(-ary interventions) within an individually tailored, multimodal lifestyle approach for people with chronic pain [114] can only be emphasized. Nevertheless, oral hygiene and its regular application are equally crucial for maintaining oral health and should be recognized alongside diet. Despite the growing evidence showing poor oral and gastrointestinal health in people with chronic pain, few treatment programs for chronic pain nowadays take diet and oral hygiene into account. This is unfortunate and allows room for improvement, as increasing scientific evidence supports both dietary and oral factors as a perpetuating factor for chronic pain [24,35,115,116,117,118]. Moreover, poor dietary habits can be considered as adverse lifestyle factors that partly account for the observed excess mortality among people with persistent pain [119,120]. Therefore, nutrition should be considered within a person-centered approach to chronic pain management [121]. A meta-analysis of the available literature concluded that an altered dietary pattern and altered specific nutrient intake may have analgesic properties for patients with persistent pain (evidence level 1a) [122]. Another systematic review concluded that plant-based diets might have analgesic effects for people with chronic pain [118]. Similarly, several studies have demonstrated the analgesic effects of a diet low in fermentable oligosaccharides, disaccharides, monosaccharides, and polyols (low-FODMAP diet) on abdominal pain in patients with irritable bowel syndrome; however, a systematic review reported that this diet induces gut microbiota changes that may be detrimental [123]. The precise action of these analgesic effects remains to be unraveled, but preclinical studies support the idea that such analgesic effects of diets arise from a potential influence on central sensitization (evidence level 5) [124,125].

Poor dietary habits often—but not always—relate to overweight or obesity [126]. Overweight and obesity are pandemics of global health concerns recognized as the leading public health problem in industrialized countries [127,128]. Meta-analyses confirm that overweight and obesity are positively associated with low back pain [126,128,129,130,131,132,133] and knee osteoarthritis [134,135], with chronic pain being more prevalent in those having a higher body mass index [136]. Moreover, overweight and obesity are associated with more severe and debilitating chronic pain: in people with chronic low back pain, pain intensity and disability show dose–response relationships to body mass index, waist circumference, percent fat, and fat mass [132]. Regarding socio-economic impact, overweight and obesity in patients with chronic pain are also related to higher rates of healthcare seeking for pain [126].

### 6.5. Additional Treatment Options

Other options for implementing oral and gastrointestinal health science in healthcare for patients with chronic pain include tailored, evidence-based oral health interventions, for instance, providing guideline-recommended, minimally invasive periodontal management for treating periodontitis [137,138] in patients with chronic pain presenting with this common oral health condition, and co-administration of gut-active supplements such as pro-, pre-, and synbiotics. A systematic review found evidence suggesting that both synbiotics and probiotics consistently reduced painkiller consumption, excluding triptan use, in people with migraine [23]. Probiotics and synbiotics were found to improve migraine frequency; synbiotics resulted in reduced migraine pain severity in both adult and pediatric migraine [23]. Similarly, in individuals with irritable bowel syndrome, probiotics and synbiotics have demonstrated modest but consistent reductions in abdominal pain and overall symptom severity [139,140]. However, most data came from pilot data that remain to be confirmed in fully powered clinical trials [23,141], and whether the observed improvements are clinically important remains to be established as well. While a comprehensive overview of studies exploring the potential of pro-, pre-, and synbiotics for improving gut health in people with chronic pain is well beyond the scope of this paper, it is striking to see that most of these studies examined only short-term (< 6 months) effects only, without any data regarding changes in the microbiome or inflammatory markers for the post-supplement washout phase.

Finally, considering the importance of common chronic pain comorbidities such as overweight/obesity and sleep disorders also implies that effective management of the relevant comorbidities can potentially benefit gut health in patients with chronic pain. Indeed, as explained above, sleep disorders such as chronic insomnia are also associated with changes in the gut microbiome [58]. For example, a significant difference in sleeping hours between working days (when social drivers determine sleep habits) and weekends plus holidays (when there are no social drivers and the person can sleep as long as she/he like) can be identified in the patient’s sleep diaries. Sleep diaries [142] can be used for assessing sleep timing on free days (i.e., when constraints on sleep timing are low) in a cost-efficient, non-invasive way [143]. Sleep on work-free days serves as a proxy for internal time as it is considered to reflect the natural sleep cycle, while workday sleep is thought to be more reflective of external, social time. The difference between workday and free day sleep timing is termed the ‘social jetlag’ [143], and can serve as an indication of circadian desynchronization. In this situation, sleep management can aim at synchronizing daily activities, including striving towards similar active hours during working and weekend days, and optimizing meal timing to match the ‘circadian reprogramming’. This way, sleep management can complement the dietary advice/intervention. More specifically, we aim for better standardized meal timing throughout the week (not only on working days). Indeed, the gut microbiota have a circadian rhythm themselves, and a preclinical study suggests that the gut microbiome regulates stress responsiveness in a circadian manner by influencing the hypothalamus–pituitary–adrenal axis [144], which might be crucial for adaptive coping with stress throughout the day. Such observations fuel best-evidence clinical practice guidelines advocating a multimodal lifestyle approach for patients with chronic pain [18], including combining sleep treatment with dietary intervention, stress management, and physical activity interventions [145].

## 7. Limitation

While this narrative review provides an overview of available evidence regarding oral and gastrointestinal health in people with chronic pain, several limitations should be acknowledged. First, this narrative review did not employ a systematic search strategy with prespecified inclusion and exclusion criteria, nor did we perform formal quality assessments of included studies. However, we conducted an extensive literature search and synthesized findings to establish consensus on the topic. Second, evidence linking the oral and gut microbiome to pain remains limited and is predominantly observational, limiting causal inference. Although animal models and preliminary fecal microbiota transplantation studies suggest a potential causal role for the gut microbiome, evidence for the oral microbiome is in its infancy and requires further investigation. While emerging data on mechanistic pathways provide biologically plausible links between oral and gut dysbiosis, systemic inflammation, and pain, well-designed longitudinal and interventional studies are needed to establish whether alterations in the oral and gut microbiome contribute causally to pain processes.

## 8. Conclusions

**Figure 4:**
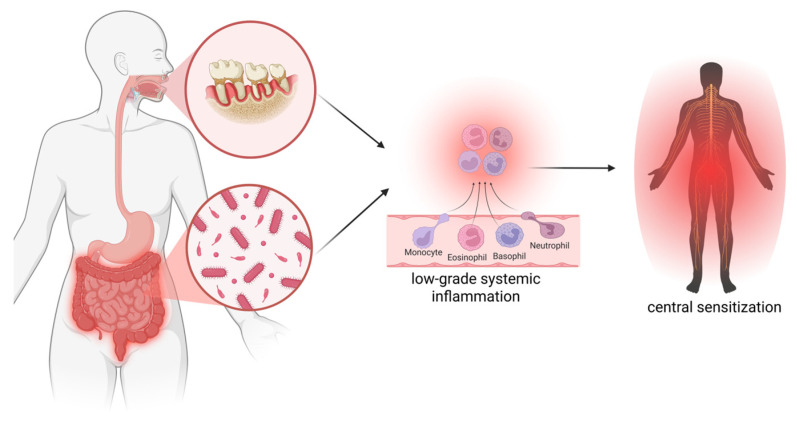
Proposed model linking oral health issues and gut dysbiosis to the pathophysiology of chronic pain: Oral health issues (e.g., periodontitis) or gut dysbiosis potentially trigger or perpetuate low-grade systemic inflammation, which in turn can trigger or perpetuate the increased sensitivity of the nervous system (‘central sensitization’) typically seen in patients with chronic pain. Note: direct evidence linking oral health or gut dysbiosis to low-grade systemic inflammation or central sensitization in people with chronic pain is currently unavailable and represents an important research priority.

Adding to our understanding of the role of low-grade, systemic inflammation and lifestyle factors in chronic pain, mounting evidence suggests an important role for gastrointestinal and oral health in people with chronic pain. Studies have revealed altered intestinal microbiota composition in various types of chronic pain, including people with chronic low back pain, knee osteoarthritis, visceral pain, fibromyalgia, tinnitus, and migraine. In addition to poor gastrointestinal health, poor oral health (e.g., periodontitis, tooth loss) is also found in different subgroups of the chronic pain population, including abdominal pain, low back pain, fibromyalgia, and rheumatoid arthritis. Such observations potentially fit our current understanding of chronic pain: both oral health issues and gut dysbiosis potentially trigger or perpetuate low-grade systemic inflammation [3,4], which in turn can trigger or perpetuate the increased sensitivity of the nervous system (‘central sensitization’) as typically seen in patients with chronic pain [5] (Figure 4). Although this notion is supported by findings showing associations between a pro-inflammatory diet and features of central sensitization in patients with chronic low back pain [146], direct evidence linking either oral health or gut dysbiosis to low-grade systemic inflammation or central sensitization in people with chronic pain is currently unavailable and represents an important research priority.

Evidence supporting a compelling role for oral and gastrointestinal health in chronic pain is growing; however, many available studies on the gut microbiome in this population are of limited quality, underscoring the need for further research using State-of-the-Art methodology to confirm and extend these findings [147]. Especially the study of the role of oral health in chronic pain is in its infancy and requires further investigation. In addition to longitudinal cohort studies examining the role of oral and gastrointestinal health in people with chronic pain, studies exploring possible (targetable) factors that affect the oral and/or gut microbiome are needed. Given the high prevalence of polypharmacy in chronic pain populations and the bidirectional interplay between the gut microbiome and commonly prescribed drugs, medication-induced dysbiosis may paradoxically perpetuate systemic inflammation and/or impair drug efficacy. This highlights the need for regular medication review, minimizing microbiome-disruptive combinations, and considering gut-supportive co-interventions as part of comprehensive pain management. However, direct evidence linking medication-induced microbiome alterations to inflammation and pain outcomes remains limited, making pharmacomicrobiomics a key research priority [104].

Fueled by the growing evidence supporting poor oral and gastrointestinal health in at least a subgroup of the chronic pain population, ways to implement oral and gastrointestinal health to improve care for people with chronic pain are presented. These include recognizing oral and gastrointestinal health issues as common chronic pain comorbidities that require tailored pain management/treatment, screening all patients with chronic pain for possible oral and gastrointestinal health issues, providing oral and gastrointestinal health science educating, considering the possible interplay between the gut microbiome and drug treatment (including polypharmacy), expanding the importance of dietary interventions, and considering the importance of comorbidities (e.g., chronic insomnia, overweight/obesity, psychological disorder) in the multimodal lifestyle management for patients with chronic pain. However, sound treatment studies examining possible treatment strategies to improve oral or gut health in people with chronic pain represent another research priority. Such randomized clinical trials can not only shed light on new, innovative ways of improving care for people with chronic pain, but also examine the possible causal link between poor oral or gut health and chronic pain. This notion also emphasizes that many of the proposals presented here for implementing oral and gastrointestinal health science in healthcare for people with chronic pain require empirical testing.

## Data Availability

No new data were created or analyzed in this study. Data sharing is not applicable to this article.
